# The Prevalence of Postpartum Depression and Its Association with Food Insecurity among Mothers Referring to Community Health Centers 

**Published:** 2018-10

**Authors:** Neda Ezzeddin, Hassan Jahanihashemi, Roza Zavoshy, Mostafa Noroozi

**Affiliations:** 1 Department of Community Nutrition, School of Nutrition Science and Food Technology, Shahid Beheshti University of Medical Sciences, Tehran, Iran.; 2 Children Growth Research Center, Qazvin University of Medical Sciences, Qazvin, Iran.; 3 Department of Nutrition, Children Growth Research Center, Qazvin University of Medical Sciences, Qazvin, Iran.

**Keywords:** *Food Security*, *Pregnancy Complication*, *Postpartum Depression*, *Socioeconomic*

## Abstract

**Objective:** Postpartum depression (PPD) is a condition which may compromise both maternal and neonatal health. The present study was conducted to determine the prevalence of PPD and its association with demographic, socioeconomic, obstetric, and household food security status.

**Method** **:** This cross sectional study was conducted in community health centers in west of Tehran. A total of 325 women were selected by stratified sampling method from community health centers. A sociodemographic questionnaire, USDA 18-item questionnaire, and Edinburgh Postnatal Depression Scale (EPDS) questionnaire were used for data collection. Data were analyzed using both descriptive and analytic analyses, such as chi-squared test and logistic regression in SPSS 22.

**Results: **The prevalence of PPD and food insecurity among the studied population was 35.4% and 34.2%, respectively. The results of this study revealed a significant association among PPD and food insecurity (OR = 6.690, CI = 3.118-14.353, p<0.001), the levels of economic satisfaction (OR = 3.419, CI = 1.241-9.420, P = 0.017), pregnancy loss (OR = 1.899, CI = 1.006-3.582, p = 0.048), and pregnancy complications (OR = 1.853, CI = 1.083-3.170, P = 0.024).

**Conclusion: **Based on the results of this study, household food insecurity may predispose mothers to PPD. Moreover, it was observed that mothers with poor economic satisfaction were more likely to be depressed. Histories of pregnancy loss and pregnancy complications were other risk factors for PPD.

Postpartum depression (PPD), whose onset is generally within 12 weeks afterdelivery, is a disorder that is often unrecognized and undertreated (1, 2). PPD is characterized by sadness, fatigue, irritability, and disinterest in life events (3). PPD may compromise both maternal and neonatal health in different ways, such as infant development, breast feeding, sleep routines, vaccinations, and safety practice (4, 5). Economic, social and biological factors, obstetrical history, lifestyle and history of mental illness are identified as risk factors for PPD (6). 

Unwanted pregnancy, due to lack of family planning, may increase the prevalence of PPD (7). Giannandrea et al. (8) found that women with the histry of loss of pregnancy (miscarriage, stillbirth, or induced abortion) were more likely to be diagnosed with psychiatric disorder, particularly depression.

Social factors, such as income inequality increases the risk of PPD (9). Studies showed that poor women experience more food insecurity compared to higher income groups (10). Household food security is defined as accessibility of enough, safe, and good-quality food for all individuals at all times to meet their dietary needs to support a healthy and productive life (11); and food insecurity refers to an opposite condition and is related to poverty and low-income (12). Food insecurity is an important problem in the developing world and has impacts on nutrition status, growth, and development (13). The association of food insecurity with maternal depression and decreased mental health status has been reported in several studies (12, 14–16). However, a few studies have assessed the association between PPD and food insecurity (17). In Iran, most studies have examined the association between PPD and mothers’ diet (18, 19) or the association between food security and maternal depression (not PPD) (20). Therefore, the present study was conducted to determine the prevalence of PPD and its related risk factors, with emphasis on food insecurity, in a small population of Iranian mothers. 

## Materials and Methods

**1.  Study Population, Design, and Data Collection**

This cross sectional study was performed from March to June 2014 in community health centers in the west of Tehran. The population of the study included postpartum women (3 to 8 months postdelivery), aged 18-45 years, with no history of depression (self-reported and according to didn’t visit a psychologist or psychiatrist for depression) and chronic disease (such as cardiovascular disease, diabetes, cancers and endocrine disease).In this study, all women agreed to participate in the study and signed the informed consent form. (The ethical code: D.44.21039).

A total of 8 community health centers in the west of Tehran, which provided a good distribution of all 16 centers based on covered population and geographic area, were randomly selected. Then, using stratified sampling technique (proportional to size), 325 women were randomly selected from these community health centers.

**2.  Measures**

*2-1. Food Security Assessment*

Food security status was evaluated using the USDA (US Department of Agriculture) questionnaire (21). This 18-item questionnaire, which assessed household food security status, was completed by interviewing women. Scoring the USDA 18-item questionnaire is as follows: responses “most of the time correct” and “sometimes correct” in questions 1 to 3 and 11 to 13, “almost every month” and “some months” in questions 5, 10, and 16, and “yes” in questions 4 and 6 to 9, 14, 15, 17, and 18 are scored 1; and responses “is not correct”, “does not know or avoids”, “only once or twice a month”, and “no” are scored 0. The final score is calculated based on the number of positive responses.

The studied women were divided into 4 groups based on the acquired scores of the questionnaire: (1) food secure, (2) food insecure without hunger, (3) food insecure with moderate hunger, and (4) food insecure with severe hunger (Table 1) (22). The 2 groups of "food insecure with moderate hunger" and "food insecure with severe hunger" were merged together and formed the food insecure with hunger group. The validity of this questionnaire has been evaluated in another study on Iranian households in Shiraz and results demonstrated acceptance of the questionnaire (23). 

*2-2. Postpartum Depression Assessment*

PPD was assessed by the Edinburgh Postnatal Depression Scale (EPDS) questionnaire. EPDS is a 10-item self-report scale for measuring the intensity of depressive symptoms experienced within the past 7 days, and each item is rated on a 4-point scale from 0 to 3. The cut-off point for PPD is 12/13 or greater. Scores of 10 to 12 represent borderline and 0 to 9 non-depressed women (24). In this study, we used the Persian version of EPDS with cut-off of 13 and reliability of 0.83 (Cronbach's alpha) (25).

*2-3. Assessment of Demographic, Socio economic, and Obstetric Status*

Demographic and socio-economic characteristics (including maternal and paternal age; education; occupational status; and level of economic satisfaction) and obstetric information (including pregnancy ranks, history of pregnancy loss: miscarriage, stillbirth, or induced abortion; unwanted infant gender; unwanted pregnancy; type of delivery; and pregnancy complications, such as gestational diabetes mellitus, pre-eclampsia, anemia, high blood pressure, nausea and vomiting, premature contractions, and bleeding) were collected via the questionnaire.

To assess the levels of economic satisfaction, mothers were asked to choose a number from 1 to 10 (subjectively) to describe their satisfaction. For data analysis, the scores were placed in 3 categories: (a) low satisfaction, 1 to 4, (b) moderate satisfaction, 5 to 7, and (c) high satisfaction, 8 to 10.

**3.  Statistical Analysis**

The association between PPD and qualitative (pregnancy ranks, type of delivery, history of pregnancy loss, unwanted infant gender, unwanted pregnancy, parents occupational status, parents educational levels, levels of economic satisfaction, pregnancy complications, and household food security status) and quantitative (maternal age) variables were evaluated with chi-squared and t tests, respectively, in SPSS22. Also, the logistic regression was used to control the variables that were statistically associated with PPD. The significance level was set at 0.05.

## Results

The mean age of participants was 28.62 ±5.67 years. Most of the mothers were housekeepers (86.5%), graduated (78.2%), and in the moderate level of economic satisfaction (64.1%). The prevalence of food insecurity and PPD among the studied women was 34.2% (n = 111) and 35.4% (n = 115), respectively. unwanted pregnancy (20.6%) and unwanted infant gender (13.7%) have also been reported, and the prevalence of vaginal delivery was very low (22.2%). More detailed information about demographic, socioeconomic, and obstetric information is presented in Table 3.

The prevalence of food insecurity was significantly higher among depressed women and it was positively associated with food insecurity scores (P< 0.001) (Table). Among the examined variables, 8 variables (maternal education (P = 0.013), paternal education (P = 0.010), father employment (P = 0.018), economic satisfaction (P< 0.001), history of pregnancy loss (P = 0.048), unwanted pregnancy (P< 0.001), unwanted infant gender (P = 0.004) and pregnancy complications (P< 0.001) were significantly associated with PPD. However, there was no significant association among other variables (maternal age (P = 0.404), paternal age (P = 0.121), maternal occupational status (P = 0.226), type of delivery (P = 0.070), and pregnancy ranks (P = 0.495)) and PPD (Table 2).

Based on the results of logistic regression analyses, food insecurity, economic satisfaction, history of pregnancy loss, and pregnancy complications were significant (P< 0.05) (Table 4).

## Discussion

The result of the study revealed a significant association among household food security status, levels of economic satisfaction, history of pregnancy loss, and pregnancy complications with PPD, but it did not have any significant association with other variables, including parents’ age, parents’ education level, parents’ occupational status, pregnancy ranks, type of delivery (NVD or caesarean), unwanted pregnancy, and unwanted infant gender.

The prevalence of PPD in developed (high-income) countries is almost 13% and the prevalence of PPD may be even greater in developing countries (7). In this study, the prevalence of PPD among mothers was 35.4%. Khooshemehry conducted a study on mothers who referred to community health centers in the north of Tehran and found PPD prevalence of 30% (26). However, in the Manshoori’s study, it was reported to be 68.5% among mothers who referred to community health centers in Rafsanjan (27), which is higher than our studied mothers. In another study by Abdollahi, the prevalence of PPD was 10.7% among mothers who referred to rural and urban primary health care centers of Mazandaran province (28). According to a meta-analysis and systematic review, the prevalence of PPD in Iran is 24.3%, based on the Edinburgh Postnatal Depression Scale; also, a significant geographic difference was observed in the prevalence of PPD (29). 

The prevalence of pregnancy complications is higher among women in developing countries than in developed countries (30). In a cohort study on 4941 pregnant women, it was shown that pregnancy complications increased the risk of PPD (31). In this study also, such an association was observed that was consistent with Shivalli and Blom studies (31, 32).

In the final logistic regression analyses, no significant association was found between unwanted pregnancy and PPD. This finding was consistent with the result of the study by Blom (31) and Sooki (33). However, in 

Barton (34) and Ria (35) studies, unwanted pregnancy was an important risk factor for PPD because it impose anticipated strain on families in meeting their basic needs (36).

The results of the present study revealed that household food insecurity is an important risk factor for PPD symptoms among mothers, which is consistent with the results of the study conducted by Dewing (17) and Patel (37). Food security is important for mothers’ health, especially during pregnancy, because nutritional demands increase in this period (12). Studies have also indicated that food insecurity leads to maternal stress during pregnancy, and food-insecure pregnant mothers worry about having enough food for themselves and their family (38). Stressful life events put women at risk for PPD (39). According to some studies, depression could be alleviated by decreasing household food insecurity (15, 40). We just assessed food security among mothers, but we did not have any data on nutritional intakes and dietary biomarkers from blood samples or the levels of mother stress during pregnancy. Thus, we could not provide the mentioned reasons in other studies for our results, and they are just possible reasons for the observed associations.

**Table 1:** Classification of the Household Food Security Status Based on Scores

**Household ** **Food Security ** **Status**	**Number of Positive ** **Responses**	
	Households without children under 18 years	Households with children under 18 years
Food secure	0-2	0-2
Food insecurity without hunger	3-5	3-7
Food insecurity with moderate hunger	6-8	8-12
Food insecurity with sever hunger	9-10	13-18

**Table 2:** The Association between Postpartum Depression and Quantitative Variables among Studied Mothers

**Variables**	**Having PPD** **Mean±SD** *			**P-value** **
	**Yes**	**No**	**Total**	
Mother age	28.97±6.37	28.42±5.25	28.62 ±5.67	0.404
Father age	33.89±6.73	32.80±5.60	33.19±6.04	0.121

* SD= standard deviation

** T test

**Table 3:** The Association between Postpartum Depression and Qualitative Variables among Studied Mothers

**Variables**	**Having PPD** **N (%)**			**P-value** *
	**Yes**	**No**	**Total**	
Pregnancy ranks 1 2 >2 Total	51(44.3) 38(33) 26(22.6) 115(100)	106(50.5) 66(31.4) 38(18.1) 210(100)	157(48.3) 104(32) 64(19.7) 325(100)	0.495
Type of delivery Nvd** Caesarean Total	19 (16.5) 96(83.5) 115(100)	53(25.2) 157(74.8) 210(100)	72(22.2) 253(77.8) 325(100)	0.07
History of pregnancy loss Yes No Total	31(27) 84(73) 115(100)	37(17.6) 173(82.4) 210(100)	68(20.9) 257(79.1) 325(100)	0.048
Unwanted infant gender Yes No Total	24(21.2) 89(78.8) 113(100)	20(9.6) 189(90.4) 209(100)	44(13.7) 278(86.3) 322(100)	0.004
Unwanted pregnancy Yes No Total	38(33) 77(67) 115(100)	29(13.8) 181(86.2) 210(100)	67(20.6) 258(79.4) 325(100)	<0.001
Mother occupational status Employment Housekeepers Total	12(10.4) 103(89.6) 115(100)	32(15.2) 178(84.8) 210(100)	44(13.5) 281(86.5) 325(100)	0.226
Father occupational status Self-Employed Government employee Total	87(76.3) 27(23.7) 114(100)	132(63.5) 76(36.5) 208(100)	219(68) 103(32) 322(100)	0.018
Education level of mother Under diploma Diploma or Higher Total	34(29.6) 81(70.4) 115(100)	37(17.6) 173(82.4) 210(100)	71(21.8) 254(78.2) 325(100)	0.013
Education Level of father Under graduated Graduate Total	39(33.9) 76(66.1) 115(100)	44(21) 166(79) 210 (100)	83(25.5) 242(74.5) 325(100)	0.010
Levels of economic satisfaction Poor Moderate Good Total	42(36.5) 65(56.5) 8(7) 115(100)	32(15.4) 142(68.3) 34(16.3) 208(100)	74(22.9) 207(64.1) 42(13) 323(100)	<0.001
Household food security status Secure Unsecure without hunger Unsecure with moderate and severe hunger Total	51(44.3) 24(20.9) 40(34.8) 115(100)	163(77.6) 32(15.2) 15(7.1) 210(100)	214(65.8) 56(17.2) 55(16.9) 325(100)	<0.001
Obstetric complications Yes No Total	62(53.9) 53(46.1) 115(100)	68(32.4) 142(67.6) 210(100)	130(40) 195(60) 325(100)	<0.001

* Chi-square test

** Normal Vaginal Delivery

**Table 4:** Final Logistic Regression Model for Predicting Postpartum Depression

**variables**	**P-value**	**OR** *	**95 % C.I** **	
			**Lower**	**Upper**
Household food security status				
Food secure		Reference		
Food insecure without hunger	0.070	1.883	0.948	3.739
Food insecure with moderate and severe hunger	<0.001	6.690	3.118	14.353
Unwanted pregnancy	0.133	1.677	0.854	3.294
Paternal education	0.466	1.341	0.610	2.945
Maternal education	0.923	1.042	0.452	2.401
Economic satisfaction				
Poor		Reference		
Moderate	0.017	3.419	1.241	9.420
Good	0.153	1.926	0.784	4.733
Unwanted infant gender	0.360	1.470	0.644	3.356
Father occupational status	0.213	1.486	0.797	2.770
Pregnancy loss	0.048	1.899	1.006	3.582
Obstetric complication	0.024	1.853	1.083	3.170

Note: table is based on results of binary logistic regression (only for significant situations

Based on chi-square test on table 3).

* Odds ratio

** Confidence Interval

## Limitation

The sample size in the study had enough statistical power to identify a significant association of PPD with household food security status, levels of economic satisfaction, and history of pregnancy loss and pregnancy complication; however, for other variables, a larger sample size may be required. In this study, we only assessed food security among mothers and we did not assess other nutritional considerations. Therefore, future studies with larger sample size and more nutritional assessments or even longitudinal studies may provide more accurate results. In this study, to better assess household food security, we assessed PPD risk factors in women with no history of referring to clinics for depression Thus, the prevalence of PPD might have been underestimated. Another limitation was that this study was conducted in a small geographic area, so we could not generalize the results to all areas.

## Conclusion

Household food insecurity may predispose mothers to PPD. We also observed that mothers with poor economic satisfaction were more likely to be depressed. The history of pregnancy loss and pregnancy complications was another risk factor for PPD.

The aim of this study was to assess PPD risk factors. By identifying PPD risk factors in each area, preventive and curative interventions will be more effective.
